# The prevalence of Black/African American individuals in concussion literature: a systematic review and meta-analysis

**DOI:** 10.3389/fpubh.2024.1430428

**Published:** 2024-08-08

**Authors:** Taia MacEachern, Ava John-Baptiste, Anita Christie

**Affiliations:** ^1^School of Kinesiology, Faculty of Health Sciences, Western University, London, ON, Canada; ^2^Department of Epidemiology and Biostatistics, Schulich School of Medicine and Dentistry, Western University, London, ON, Canada; ^3^Department of Anesthesia & Perioperative Medicine, Schulich School of Medicine and Dentistry, Western University, London, ON, Canada; ^4^Schulich Interfaculty Program in Public Health, Western University, London, ON, Canada

**Keywords:** concussion, mTBIs, prevalence, health equity, social determinants of health, disparities, Black/African American

## Abstract

**Introduction:**

Comprising approximately 13.6% of the United States population, Black/African American individuals are overrepresented in sports associated with a high risk of concussion. However, there has been a notable absence of systematic reviews examining whether concussion literature accurately reflects the participation and experiences of Black/African American individuals. Therefore, this study aims to systematically review the prevalence of Black/African American individuals compared to White individuals diagnosed with concussions in the literature.

**Methods:**

A systematic search was performed across four electronic databases: PubMed, MEDLINE (Ovid), Scopus and Web of Science. Articles were searched from inception to January 5, 2022. Prevalence data were extracted in accordance with the Preferred Reporting Items for Systematic Reviews and Meta-Analyses guidelines. A meta-analysis of proportions was conducted within hospital records and national survey data.

**Results:**

Among 447 identified studies, 11 were included, representing 1,839,901 individuals diagnosed with a concussion, with 73.6% identifying as White and 12.5% identifying as Black/African American. The mean proportion of Black/African American diagnosed with a concussion in hospital records (13.9%; 95% CI [12.8, 15.1]) exceeded that in national surveys (6.4%; 95% CI [3.5, 11.3]) but lower than sports-centered studies (16%).

**Discussion:**

These findings underscore the need to address racial disparities in healthcare within the broader context of social determinants of health and systemic inequities. By identifying gaps in the current research, this study lays the foundation for future investigation aimed at elucidating and addressing healthcare disparities.

## 1 Introduction

The exploration of social determinants of health’s influence on concussion identification, diagnosis, and rehabilitation is a growing focus in concussion literature (1). Notably, race has emerged as a significant determinant of health (2), particularly for individuals identifying as Black/African American, exerting substantial influence on these processes.

Historically, Black/African American individuals have been overlooked and excluded from medical literature (3). A recent systematic review demonstrated that only 15% of concussion literature within athletic populations over the past decade included the racial identification of participants, underscoring the deficiency in addressing race as a demographic variable within concussion literature (3). This deficiency is particularly problematic given reported racial disparities in both concussion incidence (4, 5), and outcomes (6).

Despite comprising approximately 13.6% of the United States population Black/African American individuals are disproportionately overrepresented in contact and collision sports, elevating their concussion risk (7, 8). Paradoxically, recent studies have revealed disparities in concussion diagnosis rates, with Black/African American individuals being diagnosed less frequently compared to their White counterparts (6, 9). Social inequities, including lower concussion symptom knowledge (10), and barriers to healthcare access (6, 9), are believed to contribute to these discrepancies.

Previously, a systematic review addressed the reporting of race and ethnicity in sports-related concussion (SRC) studies (3). However, SRC is only one mechanism of injury. In the pediatric population, motor vehicle collisions, and falls account for the majority of concussion injuries, followed by nonaccidental trauma and SRC (11). In older adults, fall-related head-injuries are cited as the most common concussion mechanisms (12). Broadening the scope to address the reporting of race in all concussion literature is imperative to ensure accurate understanding of the issue. Therefore, this study aims to systematically review the prevalence of Black/African American individuals compared to White individuals diagnosed with concussions in the literature. Such findings hold the potential to shed light on a demographic currently overlooked in concussion literature.

## 2 Methods

A systematic review was conducted in accordance with the Preferred Reporting Items for Systematic Review and Meta-Analysis (PRISMA) guidelines (13). This review protocol was not registered in an online database. This systematic review and meta-analysis were exempt from the institutional Research Ethics Board (WesternREM) as it involved data that is publicly available.

A literature review was conducted using the following databases: PubMed, Ovid Medical Literature Analysis and Retrieval System Online (MEDLINE), Scopus and Web of Science. The following search logic was used to extract articles from the database: (Black OR “African American”) AND (concussion OR “brain concussion” OR “mild traumatic brain injury” OR mTBI) and the following MeSH terms: (“blackness”[All Fields] OR “blacks”[MeSH Terms] OR “blacks”[All Fields] OR “black”[All Fields] OR “African American”[All Fields]) AND (“brain concussion”[MeSH Terms] OR (“brain”[All Fields] AND “concussion”[All Fields]) OR “brain concussion”[All Fields] OR “concussion”[All Fields] OR “concussions”[All Fields] OR “concussed”[All Fields] OR “concussive”[All Fields] OR “brain concussion”[All Fields] OR “mild traumatic brain injury”[All Fields] OR “mTBI”[All Fields]). Filters within the database were set to peer-reviewed journal articles published from the databases inception up to and including articles published January 5, 2022.

### 2.1 Literature search and study selection criteria

Inclusion criteria for this review were (i) original research (including randomized clinical trials (RCT), quasi-experimental designs, case studies and series, prospective and retrospective studies, cohort studies, and pilot studies), (ii) include human participants who have sustained a concussion or mild traumatic brain injury (mTBI), (iii) include results that compare study outcomes between Black/African American and Caucasian/White/ (iv) completed in the United States of America. Articles published in English, with any sample size were included. There were no limitations to the age of participants or mechanism of injury. Diagnosis of a concussion could have been rendered at any time. Review articles and articles published in only abstract form were excluded.

The screening, full-text review and data extraction were performed by two authors (TM and AC) using Covidence Systematic Review software (Veritas Health Innovation). TM and AC met frequently to discuss coding within the review. When a disagreement occurred, discussions continued until the two authors reached a consensus. Phase I applied the search terms to extract the first round of articles from PubMed (*n* = 131), MEDLINE (*n* = 66), Scopus (*n* = 107) and Web of Science (*n* = 133). Phase II involved preliminary distillations of the identified articles through title and abstract screening by removing studies irrelevant to the research question (*n =* 164), studies containing the wrong patient population (*n =* 19), studies containing the wrong outcome due to not comparing Black/African and White individuals (*n =* 15), focused on other areas of health (*n =* 2) and not completed in the United States (*n =* 1). Consequently, these articles were not considered for this review. During phase III, the refined list of articles (*n =* 11) was reviewed, and the following data was extracted: type of study, location, duration, funding sources, population characteristics (population type, sample size, race, age, sex), definition of concussion, number of individuals with a concussion based on racial categories, concussion assessment(s) and key findings on outcome measures related to neurocognitive assessments, and/or symptom scales. Studies reporting the prevalence or incidence of concussion in Black/African American individuals, or provided sufficient data to permit calculation, were included.

### 2.2 Statistical analysis

The proportion of Black/African American vs. White individuals with a concussion were compared using a meta-analysis of proportions. A separate random effects meta-analysis was performed for prevalence estimates derived from hospital records (5, 14–21), those derived from national survey data (22), and those generated from the medical records of athletes attending a sport concussion center (6). For the national survey data the authors were contacted and provided values for the unweighted number of individuals who reported a concussion, which were used in the meta-analysis (22). In each meta-analysis, the mean proportion of Black/African American individuals with a concussion, weighted by variance, was computed, in addition to the 95% confidence interval [CI].

### 2.3 Quality assessment and risk of bias

To assess the risk of bias in studies, the JBI Critical Appraisal Checklist tools created by International Joanna Briggs Institute were used. The JBI critical appraisal checklist was designed to determine the extent to which a study has addressed the possibility of bias in its design, conduct, and analysis, and to assess the methodological quality of a study (21). There were 10 questions to guide the evaluation of case series studies, eight for the evaluation of analytic cross-sectional studies and nine for the evaluation of prevalence studies. The questions were quantified using scores from 0 to 1. One point is given if the answer is “YES” and zero points if the answer is “NO,” “not clear” or “not applicable.”

## 3 Results

**Figure 1:**
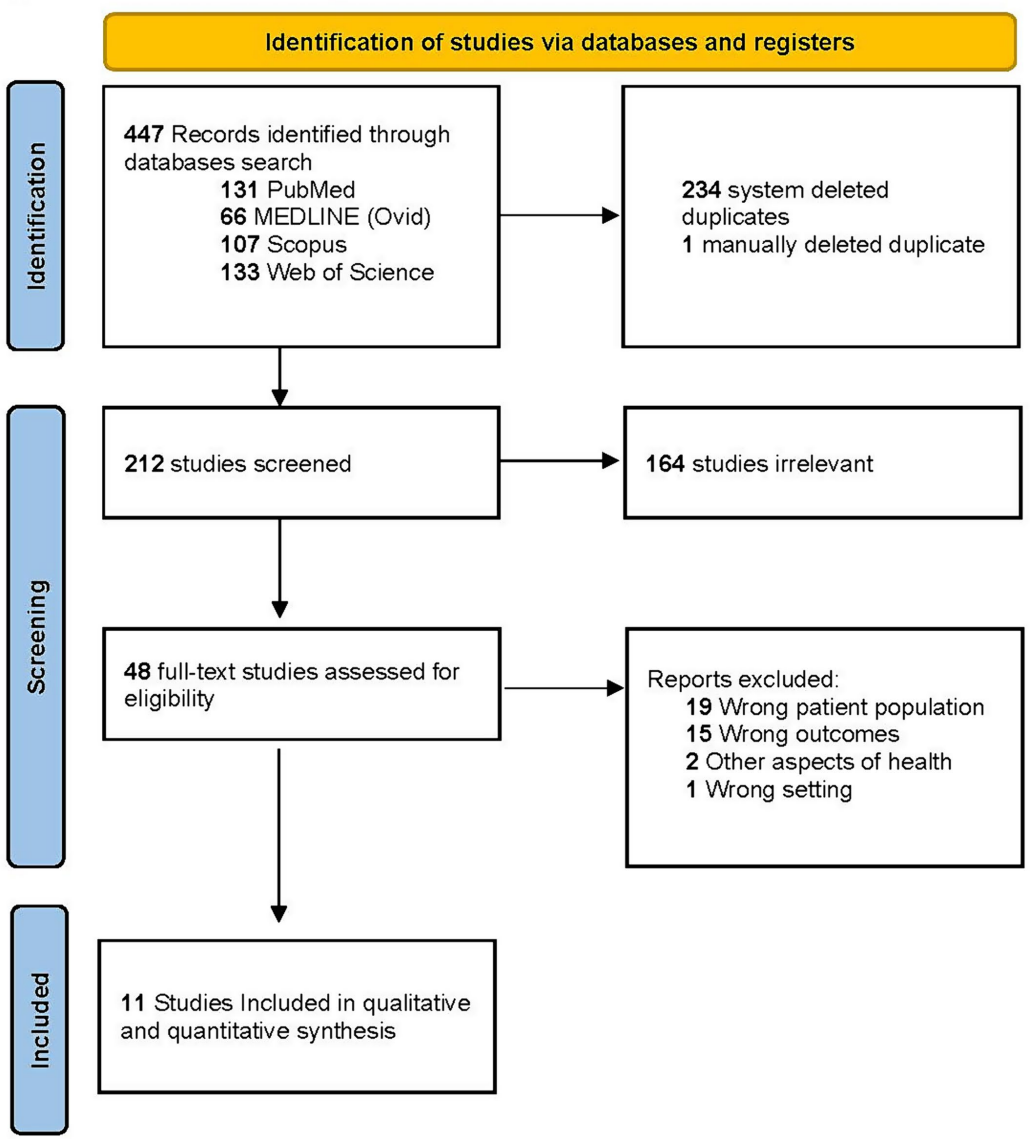
PRISMA flow diagram of study selection. Flow diagram of search results and review of studies for inclusion.

The data extraction process resulted in 11 distinct studies (5, 6, 14–22), that satisfied the predefined inclusion and exclusion criteria (Figure 1). In total, these studies encompassed 1,839,901 participants diagnosed with concussions. Among these participants, 285,014 identified as Black/African American, 1,446,999 as White and 105,740 as belonging to other racial groups (Table 1).

**Table 1:** Populations of studies included in the meta-analysis.

Study	Study type	Total	Black/African American	White	Other	Proportion Black/African American	SD proportion Black/African American
Bazarian et al., 2000 (20)	Hospital admission	71	7	60	4	0.10	0.04
Bazarian et al., 2003 (21)	Hospital admission	1,367,101*	219,329	1,096,255	51,517	0.16	0.0003
Ganti et al., 2015 (14)	Hospital admission	2,567	419	NR	NR	0.16	0.01
Holmes et al., 2016 (5)	Hospital admission	1,429	170	1,146	113	0.12	0.01
Lemme et al., 2020 (19)	Hospital admission	2,079	171	1,134	774	0.08	0.01
Lyons et al., 2019 (18)	Hospital admission	458,816*	64,124	342,904	51,788	0.14	0.0005
Rhame et al., 2021 (17)	Hospital admission	174	32	136	6	0.18	0.03
Wallace and Mannix, 2021 (16)	Hospital admission	1,263	233	739	291	0.18	0.01
Wallace et al., 2020 (15)	Hospital admission	974	120	834	20	0.12	0.01
Haarbauer-Krupa et al., 2021 (22)^a^	National survey	2,199	82	1,758	359	0.04	0.0040
Haarbauer-Krupa et al., 2021 (22)^b^	National survey	695	54	469	172	0.08	0.0102
Haarbauer-Krupa et al., 2021 (22)^c^	National survey	1,951	177	1,078	696	0.09	0.0065
Wallace et al., 2021 (6)	Athlete population	582	96	486	0	0.16	0.02

^*^Represents a weighted population.

^a^National Survey of Children’s Health (NSCH).

^b^National Health Interview Survey (NHIS).

^c^Monitoring the Future (MTF).

The age distribution varied across the 11 articles. Two studies focused solely on children and adolescents (≤18 years) (18, 22), while one study exclusively involved adults (>18 years) (17). The remaining seven studies included both child and adult participants (Table 2) (5, 6, 15, 16, 19–21). Notably, one study did not specify the age range of its participants (14).

**Table 2:** Descriptive information of included studies.

Study	Definition of concussion	Age range (year)	How were surveys conducted?	Name of racial categories	Why was race collected	Who answered survey containing race data?
Bazarian et al., 2000 (20)	Blow to the head; <10-min loss of consciousness or amnesia; GCS of 15; no new focality; no evidence of skull fracture	16–71	Twenty-five minute, neurobehavioural test battery, administered by one of four patient enrollers, each trained by a neuropsychologist. Follow up with physician after 1–2 months	African American; Caucasian; Other	Part of demographic variables (age, sex, race)	NA
Bazarian et al., 2003 (21)	Brief loss of consciousness or amnesia; GCS of 13–15; no skull fracture; non-focal neurological examination	0–99	Extraction of ICD-9 codes 800.0, 800.5, 801.0, 801.5, 803.0, 803.5, 804.0, 804.5, 850.0, 850.1, 850.5, 850.9, 854.0, and 959.01	African American/Black; White; Native American/Alaska Native; Native Hawaiian/Other Pacific Islander	The main objectives included racial comparisons	NA
Ganti et al., 2015 (14)	GCS of 13 or greater	NA	Data entry personnel were trained on the REDCap system; Extraction of ICD-9 codes 800.0–804.9, 850.0–854.1, 959.01, and 995.55	Black; Native American; Hispanic; Native Hawaiian/Pacific Islander; White	NA	NA
Haarbauer-Krupa et al., 2021 (22)^a^	NA	3–17	Self-administered web or paper questionnaire	Black, non-Hispanic; White, non-Hispanic; Hispanic; Other, non-Hispanic	Included as demographic characteristics	Parent or primary caregiver
Haarbauer-Krupa et al., 2021(22)^b^	NA	3–17	In person household interview by trained interviewers using computer-assisted personal interviewing	Black, non-Hispanic; White, non-Hispanic; Hispanic; Other, non-Hispanic	Included as demographic characteristics	Parent or primary caregiver
Haarbauer-Krupa et al., 2021 (22)^c^	NA	13–17	Paper questionnaire	Black, non-Hispanic; White, non-Hispanic; Hispanic; Other non-Hispanic	Included as demographic characteristics	Self-reported
Holmes et al., 2016 (5)	NA	2–19	Extraction of chart information if participants were diagnosed with concussion or TBI between 2007 and 2014.	African American/Black; White; Some Other Race	The primary objectives included racial comparisons	Self-reported
Lemme et al., 2020 (19)	NA	4–62	Extraction of information from all head injuries from 2012 to 2016, diagnosed as a concussion (code 52) or internal head injury (code 62), which occurred during boxing (code 1207), Martial Arts (code 3257), or wrestling (code 1270)	Black; American Indian; Asian; Hawaiian/Pacific Islander; Hispanic; White; Not Specified	NA	NA
Lyons et al., 2019 (18)	NA	7–18	Records are reviewed at the end of each day by study research coordinators and coded according to the NEISS coding manual; ED visits were categorized into the following final diagnoses: concussions, fractures/dislocations, sprains/strains/contusion, internal injuries, and other	Black; Asian; Hispanic; Non-Hispanic White; Other	The main objectives included racial comparisons	NA
Rhame et al., 2021 (17)	Sustained an isolated head injury with a GCS 14 or 15 may be included in the ED mild TBI observation protocol. Patients may have a normal or abnormal head CT.	26–68	Standardized case report forms were used by medical student, resident, or attending data abstractors to document demographic information, mechanism of injury, head CT findings based on the attending radiologist read (positive or negative for hemorrhage, types of hemorrhage, change in serial head CTs, and if the CT met imaging guideline criteria), anticoagulant medications, clinical examination (GCS), comorbidities, other injuries, reason for admission after the OU protocol in instances in which a subject required hospitalization, and disposition of all subjects admitted to the OU	African American/Black; Asian or Indian; Latino; White	NA	NA
Wallace et al., 2021 (6)	Based on the ICD-9 and 10 codes for concussion and variants as well as post-concussion syndrome	12–23	Manual chart review was performed with data extraction from provider notes into a secure REDCap database; Extraction of ICD-9 and 10 codes for concussion and variants as well as post-concussion syndrome; provider confirmed an SRC diagnosis based on the most recent Concussion in Sport Group guidelines	Black; White	The primary objectives included racial comparisons.	Self-reported
Wallace and Mannix, 2021 (16)	ICD-9-CM diagnosis codes: 850.0–850.9, 850.11, 850.12. or 959.01 “head injury not specified”	0–19	Paper-based questionnaire collected by specialty trained interviewers in 2010–2011. Years 2012–2015 were computerized questionnaires; Extraction of ICD-9-CM: 850.0–850.9, 850.11, 850.12 or 959.01 (“head injury not specified”)	Non-Hispanic Black; Non-Hispanic White; Hispanic; Other	Part of demographic variable (age, sex, race, ethnicity)	NA
Wallace et al., 2020 (15)	ICD-9-CM; GCS scores, which ranged from 13 to15, are categorized as a mild head injury, and scores ranging from 9 to 12 were categorized as moderate head injury, and 8 or less were categorized as severe.	13–19	Extraction of ICD-9-CM codes 850.9, 850.5, 850.12, 850.11, 850.0, 310.2, 850.5, 850.0	Black/ African American; White/ Caucasian	The purpose included racial comparisons.	NA

GCS, Glasgow Coma Scale; NA, not available; ICD-9-CM, International Classification of Diseases Ninth Revision Clinical Modification; TBI, traumatic brain injury; REDCap, Research Electronic Data Capture; NEISS, National Electronic Injury Surveillance System; CT, Computed Tomography; OU, Observation Unit; SRC, sport related concussion.

^a^National Survey of Children’s Health (NSCH).

^b^National Health Interview Survey (NHIS).

^c^Monitoring the Future (MTF).

Racial demographics were captured through various means. Some studies included racial categories as part of their demographic variables (15, 16, 20, 22), while others focused on race as a primary variable of interest (5, 6, 18, 21). However, in three studies, the rationale for collecting racial data was not explicitly states (14, 17, 19). Participants or their parent/caregiver provided information on race (5, 6, 22), though in several instances it was not stated who reported this data (Table 2) (14–21).

### 3.1 Quality assessment and risk of bias

The methodological quality was assessed using various versions of the JBI checklist. The JBI checklist for case series was used for seven studies (Table 3) (5, 6, 12, 13, 15, 18, 19), the JBI checklist for analytic cross-sectional studies was used for three studies (Table 4) (14, 16, 17), and the JBI checklist for prevalence studies was used for one study (Table 5) (20).

**Table 3:** Results of the JBI case series checklists.

	Q1	Q2	Q3	Q4	Q5	Q6	Q7	Q8	Q9	Q10	Total yes
Bazarian et al., 2000 (20)	Yes	Yes	Yes	No	No	Yes	No	NA	No	Yes	5/10
Bazarian et al., 2003 (21)	Yes	Yes	Yes	Yes	Yes	Yes	No	NA	Yes	Yes	8/10
Ganti et al., 2015 (14)	Yes	Yes	Yes	Yes	Yes	No	Yes	NA	No	Yes	7/10
Holmes et al., 2016 (5)	Yes	Yes	Yes	Yes	Yes	Yes	No	yes	No	Yes	8/10
Rhame et al., 2021 (17)	Yes	Yes	Yes	Yes	Yes	Yes	No	NA	Yes	Yes	8/10
Wallace et al., 2020 (15)	Yes	Yes	Yes	Yes	Yes	Yes	No	NA	Yes	Yes	8/10
Wallace et al., 2021 (6)	Yes	Yes	Yes	No	No	Yes	No	NA	No	Yes	5/10

NA, Not applicable.

Q1. Where there clear criteria for inclusion in this case series?

Q2. Was the condition measured in a standard, reliable way for all participants included in the case series?

Q3. Were valid methods used for the identification of the condition for all participants included in the case series?

Q4. Did the case series have consecutive inclusion of participants?

Q5. Did the case series have complete inclusion of participants?

Q6. Was there clear reporting of the demographics of the participants in the study?

Q7. Was there clear reporting of clinical information of the participants?

Q8. Were the outcomes or follow-up results of cases clearly reported?

Q9. Was there clear reporting of the presenting sites’/clinics’ demographic information?

Q10. Was statistical analysis appropriate?

**Table 4:** Results of the JBI analytic cross-sectional checklists.

	Q1	Q2	Q3	Q4	Q5	Q6	Q7	Q8	Total yes
Lemme et al., 2020 (19)	Yes	Yes	Yes	Unclear	NA	NA	Yes	Yes	5/10
Lyons et al., 2019 (18)	Yes	Yes	Unclear	Yes	Unclear	Unclear	Yes	Yes	5/10
Wallace and Mannix, 2021 (16)	Yes	Yes	Yes	Yes	No	Unclear	Yes	Yes	6/10

NA, Not applicable.

Q1. Were the criteria for inclusion in the sample clearly defined?

Q2. Were the study subjects and the setting described in detail?

Q3. Was the exposure measured in a valid and reliable way?

Q4. Were objective, standard criteria used for measurement of the condition?

Q5. Were confounding factors identified?

Q6. Were strategies to deal with confounding factors stated?

Q7. Were the outcomes measured in a valid and reliable way?

Q8. Was appropriate statistical analysis used?

**Table 5:** Results of the JBI prevalence checklists.

	Q1	Q2	Q3	Q4	Q5	Q6	Q7	Q8	Q9	Total yes
Haarbauer-Krupa et al., 2021 (22)^a^	Yes	Yes	Yes	No	Yes	Unclear	Unclear	Yes	Yes	6/10
Haarbauer-Krupa et al., 2021 (22)^b^	Yes	Yes	Yes	No	Yes	Unclear	Unclear	Yes	Yes	6/10
Haarbauer-Krupa et al., 2021 (22)^c^	Yes	Yes	Yes	No	Yes	Unclear	Unclear	Yes	Yes	6/10

NA, Not applicable.

^a^National Survey of Children’s Health (NSCH).

^b^National Health Interview Survey (NHIS).

^c^Monitoring the Future (MTF).

Q1. Was the sample frame appropriate to address the target population?

Q2. Were study participants sampled in an appropriate way?

Q3. Was the sample size adequate?

Q4. Were the study subjects and the setting described in detail?

Q5. Was the data analysis conducted with sufficient coverage of the identified sample?

Q6. Were valid methods used for the identification of the condition?

Q7. Was the condition measured in a standard, reliable way for all participants?

Q8. Was there appropriate statistical analysis?

Q9. Was the response rate adequate, and if not, was the low response rate managed appropriately?

Applying the JBI checklist for case series scores ranged from 5 to 8 out of 10 with a majority of articles scoring 8/10 (57%). Within this quality assessment only one study included follow-up results, meeting criteria for question 8, making it not applicable in all other studies. However, all studies included clear criteria (question 1), the condition to be measured in a standard, reliable way (question 2), with valid methods (question 3), and appropriate statistical analysis (question 10).

Within the three studies analyzed using the JBI analytic cross-sectional studies checklist the scores two studies (18, 19), scored 5/10 while one study (16), scored 6/10. The one study (22), that was analyzed using the JBI prevalence checklist scored a 6/10.

### 3.2 Prevalence

Where a single study encompassed multiple populations, separate proportion were calculated for each population (22). Within the meta-analysis, conducted using weighted proportions across all studies, the predominant racial identification was White, comprising 73.6% of the sample population, while Black/African American individuals constituted a smaller percentage at 12.5% among those diagnosed with a concussion.

**Figure 2:**
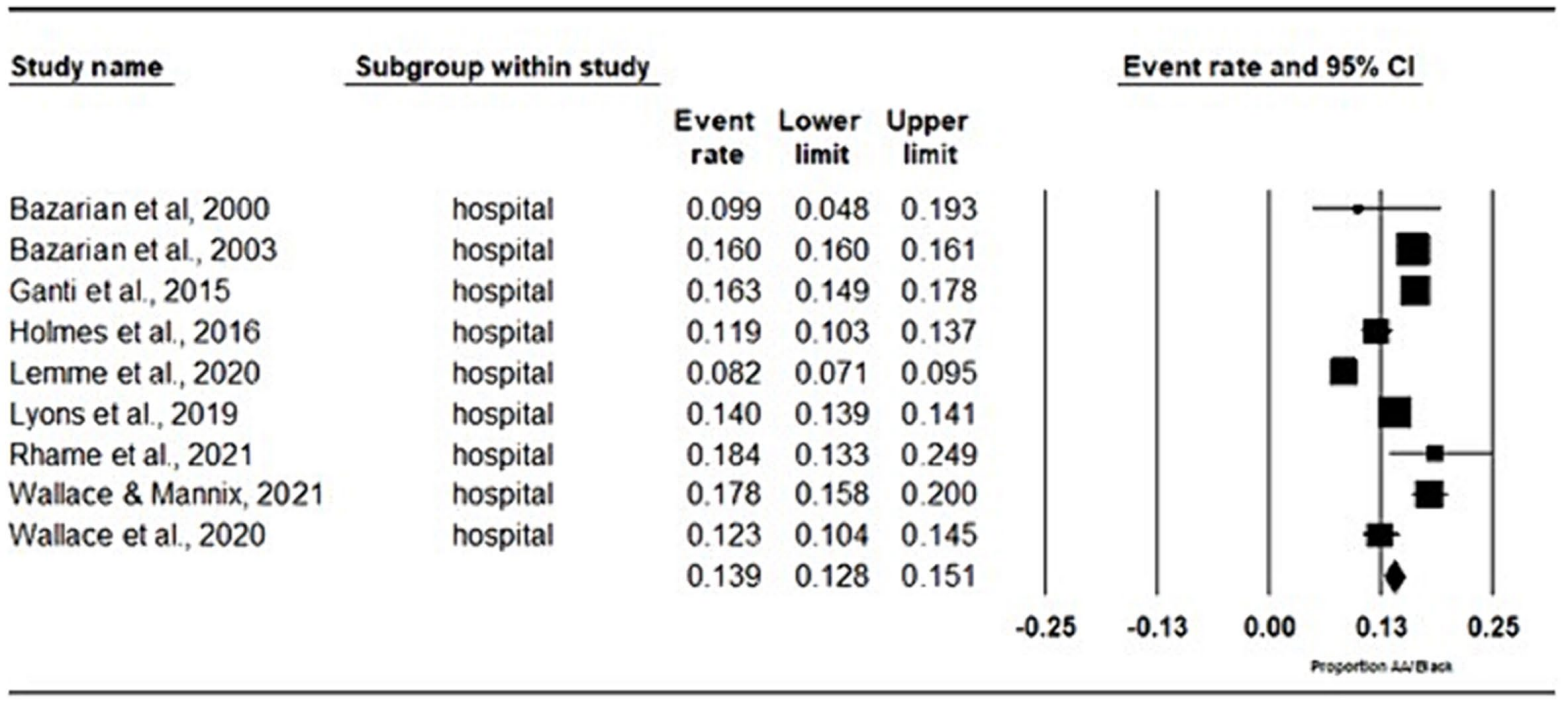
Meta-analysis of proportions of hospital/emergency department records. Proportion of AA/Black individuals included in studies that took place in a hospital and emergency department setting.

**Figure 3:**
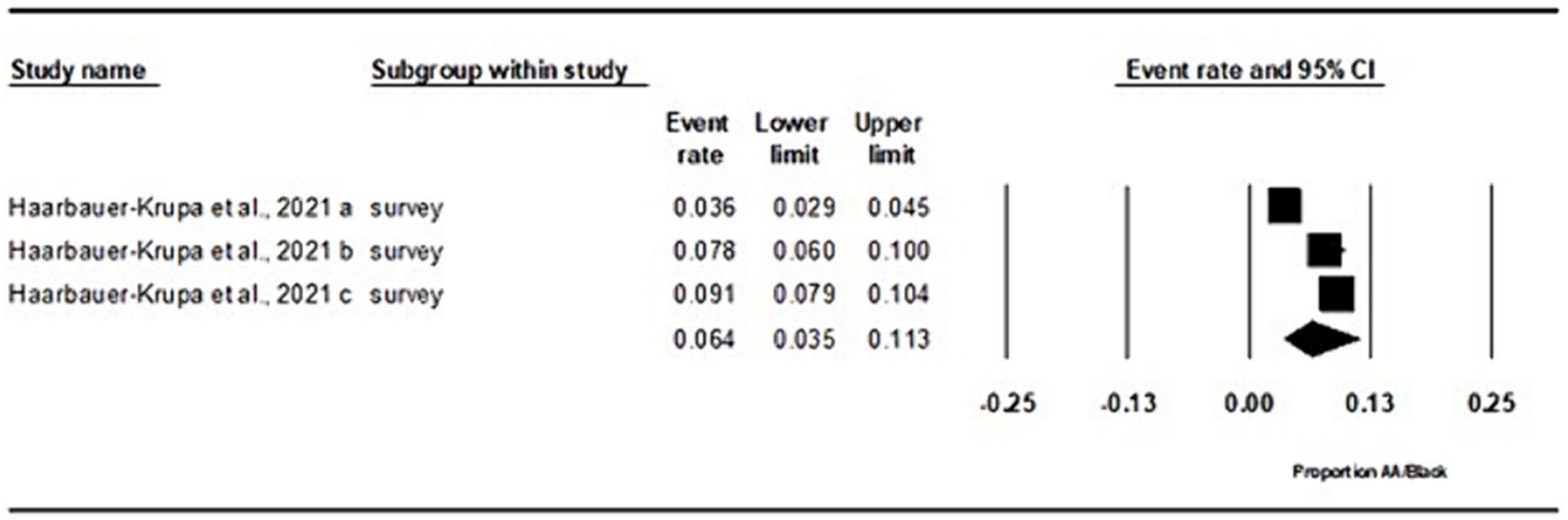
Meta-analysis of proportions of national survey data. Proportion of AA/Black individuals included in studies that collected data from a National Survey Data.

The majority of the studies (*n* = 9) included data from individuals diagnosed with a concussion in either a hospital setting or an emergency department (ED) (5, 14–21). Across these studies, the mean proportion of Black/African American individuals (13.9, 95% CI [12.8, 15.1]; Figure 2) was lower compared to those who identified as White. One study analyzed data from three distinct national survey datasets (22). Across these three datasets, a similar trend was observed, with a lower proportion of Black/African American individuals (6.4, 95% CI [3.5, 11.3]; Figure 3) compared to those identifying as White among those diagnosed with concussions. Additionally, one study focused on athletes, accessing medical records from a sport concussion center, where 16% of the concussed group identified as Black/African American (Table 1) (6).

## 4 Discussion

The influence of social determinants of health, such as race, on the identification, diagnosis, and rehabilitations of concussion is increasingly gaining attention in concussion research. In this study, the aim was to systematically review the prevalence of concussions among Black/African American individuals compared to White individuals in the existing literature. Our findings revealed variability in the representation of Black/African American individuals depending on the method of data collection. When examining hospital records, the prevalence of concussions among Black/African American individuals (13.9%) closely mirrored their representation in the general population (13.6%) (7). However, disparities emerged when comparing to national survey data, where Black/African American individuals were underrepresented at 6.4%. Conversely, in sport-related contexts, they were overrepresented, comprising 16% of diagnosed cases.

According to the 2021 census data, the racial distribution in the United States shows Black/African American individuals constitute 13.6% of the population, while White individuals make up 75.8% (7). If Black/African American individuals face similar likelihoods of sustaining concussion and have equitable access to appropriate medical care, we would anticipate their representation among concussion cases to align with their population percentage, as observed in hospital-based studies.

This alignment with population demographics may be influenced by the structure of the healthcare system in the United States. Data from 2020 indicated that 19.5% of Black individuals in the United States live below the poverty line, a rate significantly higher than the national average of 11.4% and more than double that of White individuals at 8.2% (23). Arbogast et al., (24) indicated that non-Hispanic Black patients and those with Medicaid coverage are more likely to utilize the emergency department (ED) setting as an initial point of entry into the healthcare system. However, accurate clinical identification of a concussion can be difficult due to varying definitions and criteria (25). Variations in concussion symptom presentation and lack of structural abnormalities on neuroimaging often lead concussions to be misdiagnosed in the ED (26, 27). It has previously been reported that 45% of children (26) and 56% of adults (28) that present to the ED met diagnosis criteria for a concussion, yet did not receive a diagnosis. The prevalence of misdiagnosis in the ED makes it possible that concussion clinics may be a more appropriate place to receive an accurate concussion diagnosis. However, concussion clinics are not utilized by all parts of the general population, specifically it has been found that non-Hispanic White individuals are more likely to utilize a concussion clinic and those not on Medicaid or state Child Health insurance plans were less likely to present in a specialized clinic (29, 30).

In additional to misdiagnosis, ED usage may be affected by factors such as accessibility, individual interpretation of injury severity and perception of primary care’s ability to handle acute injury and illness (31), all which have been shown to be associated with race, leading to a lower likelihood of Black/African American individuals seeking care (32–35). Additionally, previous studies have indicated disparities in concussion diagnosis rates between Black/African American individuals and White/non-Hispanic individuals within ED settings (15). Systemic factors, including implicit bias among healthcare practitioners, have been extensively studied (34, 36–38). Research has demonstrated that clinicians often exhibit moderate to strong implicit bias against Black patients (34), which can directly influence treatment recommendations and care decisions (38). Therefore, while our finding of Black/African American individuals accounting for 13.9% of concussions diagnosed in hospitals align with the distribution of the general population, it may not fully capture the concussion prevalence of Black/African individuals due to potential biases and disparities in healthcare delivery.

In the national survey data, Black/African American individuals were found to be underrepresented compared to their distribution in the population (7). These surveys primarily focused on children and adolescents, addressing lifetime prevalence, which could potentially explain the lower observed prevalence rate among Black/African American individuals. As a concussion diagnosis is based on symptom presentation, it is crucial for individuals or caregivers to possess a comprehensive understanding of concussion symptoms and seek appropriate medical attention. Previous studies have indicated a disparity in concussion symptom knowledge between Black/African American and White athletes (10, 33), as well as among parents/guardians (32).

The non-specific nature of concussion symptoms poses a risk of misdiagnosis, as many symptoms can overlap with those of other health conditions (39). Additionally, limitations related to health literacy may contribute to this underrepresentation, as medical terminology used in symptom checklists might hinder individuals from accurately identifying symptoms. Previous research has highlighted that Black/African American individuals may have lower health literacy compared to their White counterparts (40, 41). Symptoms such as “fatigue” and “nausea” for instance, may pose challenges for those less familiar with medical terminology (33). Systemic institutional factors, such as chronic underfunding of schools in Black/African American communities, may further exacerbate disparities in health literacy (42). This lower health literacy has been associated with adverse health outcomes, poorer health status and decreased satisfaction with healthcare services (40, 41).

The proportion of Black/African American individuals diagnosed with a concussion in athletic literature exceeded their representation in the general population. This observation can be attributed, in part, to the demographic composition of contact and collision sports. In a nationally representative sample, it has been reported that African American youth make up approximately 27% of participants in tackle football, while White youth make up only approximately 10% (43). Black/African American individuals comprise approximately 50% of collegiate athletes in the National Collegiate Athletic Association (NCAA) participating in high-concussion risk, revenue-producing sports such as football, men’s basketball and women’s basketball (4). This overrepresentation of Black/African American players is also seen in the NFL, with Black/African American players comprising over 70% (44). Given this significant overrepresentation in sports associated with elevated concussion risk, and the potential for athletes to have immediate access to healthcare professionals such as Athletic Trainers and Sport Medicine Physicians, that may be more familiar with concussion injuries, it is expected that the proportion of Black/African diagnosed with concussions within athlete populations would surpass that of the general population.

While variations in concussion diagnosis were evident across different forms of data collection, it is plausible that none of the findings in the concussion literature accurately reflect the Black/African American experience with concussions. Out of the 11 articles in this study, 10 relied on medical chart reviews based on a concussion diagnosis made by a healthcare professional. However, the lack of concise definitions of concussion led to nearly every study utilizing a different definition (25). Often, it was not clarified whether clinicians distinguished between a diagnosis of concussion, traumatic brain injury, sport-related concussion, or other head injuries, as the interpretation of the concussion definition was left to individual clinicians. This ambiguity, coupled with disparities in the quality and standard of care received by Black communities (35, 36, 38), may contribute to underdiagnosis within minority communities.

This notion is supported by literature contradicting the findings of this systematic review, which indicates that Black/African American individuals are underrepresented in the ED for head injuries and are less likely to be diagnosed with a concussion compared to their white counterparts (15, 18). Furthermore, feelings of distrust towards healthcare practitioners, along with discomfort and fear of discrimination experienced by Black/African American individuals, have been found to lead to delays in seeking medical care (34, 35). Given the nature of concussion diagnosis, such a delay may contribute to an under-reporting and underdiagnosis of concussions in Black/African American individuals.

### 4.1 Limitations

This review is subject to several limitations that warrant consideration. While the authors aimed to comprehensively identify and extract all articles meeting the inclusion criteria, the search was limited to four databases: PubMed, MEDLINE (Ovid), Scopus and Web of Science. These restrictions may have inadvertently resulted in the oversight of additional concussion studies.

Furthermore, the absence of a universally accepted definition of concussion poses a challenge (35). In the United States, the American Medical Society for Sport Medicine (AMSSM) released a definition of concussion in 2013 (36), updated in 2019 (37). Globally, the International Conference on Concussion in Sport revises their definition every 4 years, with updates occurring between 2001 and 2016 (38, 39). Given that the papers included in this review spanned from 2000 to 2021, changes in concussion definitions during this 21-year period may have influenced concussion recognition and diagnosis across studies. The authors acknowledge this discrepancy, however, while some agreement exists among various concussion definitions, individual diagnosis and management decisions remain within the realm of clinical judgment and therefore limiting the timeline of which this review covered had the potential to influence the results of the review based on the authors personal interpretation on the appropriate definition and diagnosis of concussion. Additional limitations stem from variable sample compositions, including variations in age demographics, and the retrospective nature of some studies. Moreover, many studies failed to provide information on potentially confounding variables such as socioeconomic status, prior concussion history, presence of ADHD, and sex, all of which have been shown to influence concussion reporting behavior and diagnosis (40–45).

## 5 Conclusion

The findings of this systematic review and meta-analysis provide valuable insight into the intricate interplay between race and healthcare, emphasizing the complexities involved in diagnosing concussions and accurately representing them in the literature. Contrary to prior assumptions, the observed findings challenge the notion of overrepresentation of Black/African American individuals in all concussion studies. These findings may be attributed to various factors stemming from social determinants of health, perpetuated by racial disparities in healthcare. However, it is essential to contextualize these healthcare disparities within the broader context of racial inequities embedded within laws, regulations, and social institutions (45).

Achieving an accurate representation of concussion is paramount. Addressing healthcare disparities requires a comprehensive understanding of the issue, and the findings of this systematic review underscore an underexplored area within healthcare research. Future research endeavors should focus on identifying health disparities associated with race that hinder Black/African American individuals from accessing appropriate care. By pinpointing gaps in the current literature, this study lays the foundation for further research aimed at elucidating and ultimately mitigating racial disparities in concussion diagnosis and care. Through efforts aimed at addressing these disparities, we can work towards ensuring equitable healthcare access and outcomes for all individuals, regardless of race or ethnicity.

## Data availability statement

The original contributions presented in the study are included in the article/supplementary material, further inquiries can be directed to the corresponding author.

## Author contributions

TM: Conceptualization, Data curation, Formal analysis, Funding acquisition, Investigation, Methodology, Validation, Writing – original draft, Writing – review & editing. AJ-B: Data curation, Formal analysis, Investigation, Methodology, Validation, Writing – review & editing. AC: Conceptualization, Data curation, Formal analysis, Funding acquisition, Investigation, Methodology, Supervision, Validation, Writing – original draft, Writing – review & editing.
